# GLT-1 Transport Stoichiometry Is Constant at Low and High Glutamate Concentrations when Chloride Is Substituted by Gluconate

**DOI:** 10.1371/journal.pone.0136111

**Published:** 2015-08-24

**Authors:** Anatoli Y. Kabakov, Paul A. Rosenberg

**Affiliations:** F. M. Kirby Neurobiology Center and Department of Neurology, Children’s Hospital and Harvard Medical School, Boston, Massachusetts, United States of America; University of Cambridge, UNITED KINGDOM

## Abstract

Glutamate is the major excitatory neurotransmitter, but prolonged exposure even at micromolar concentrations causes neuronal death. Extracellular glutamate is maintained at nanomolar level by glutamate transporters, which, however, may reverse transport and release glutamate. If and when the reverse occurs depends on glutamate transport stoichiometry (GTS). Previously we found that in the presence of chloride, the coupled GLT-1 glutamate transporter current and its relationship to radiolabeled glutamate flux significantly decreased when extracellular glutamate concentration increased above 0.2 mM, which implies a change in GTS. Such high concentrations are feasible near GLT-1 expressed close to synaptic release site during excitatory neurotransmission. The aim of this study was to determine GLT-1 GTS at both low (19–75 μM) and high (300–1200 μM) glutamate concentration ranges. GTS experiments were conducted in the absence of chloride to avoid contributions by the GLT-1 uncoupled chloride conductance. Mathematical analysis of the transporter thermodynamic equilibrium allowed us to derive equations revealing the number of a particular type of ion transported per elementary charge based on the measurements of the transporter reversal potential. We found that GLT-1a expressed in COS-7 cells co-transports 1.5 Na^+^, 0.5 Glu^-^, 0.5 H^+^ and counter-transports 0.6 K^+^ per elementary charge in both glutamate concentration ranges, and at both 37°C and 26°C temperatures. The thermodynamic parameter *Q*_*10*_ = 2.4 for GLT-1 turnover rate of 19 s^-1^ (37°C, -50 mV) remained constant in the 10 μM–10 mM glutamate concentration range. Importantly, the previously reported decrease in the current/flux ratio at high glutamate concentration was not seen in the absence of chloride in both COS-7 cells and cultured rat neurons. Therefore, only in the absence of chloride, GLT-1 GTS remains constant at all glutamate concentrations. Possible explanations for why apparent GTS might vary in the presence of chloride are discussed.

## Introduction

Glutamate is the major excitatory neurotransmitter of the central nervous system, but exposures to even low glutamate concentrations are toxic to neurons [1,2]. The only known clearance mechanism for glutamate is provided by five excitatory amino acid transporters (EAAT1-5) [3]. GLT-1, the rodent analog of human EAAT2, is the major glutamate transporter of the brain. GLT-1 knockout mice die from intractable epilepsy several weeks after birth and have only about 5% of wild-type levels of glutamate uptake activity in cortical synaptosomes [4]. Selective inhibition of GLT-1 by intrahippocampal microinjection of a specific inhibitor (WAY-855, 200–300 nmol) in rat *in vivo* killed the majority of both CA1–4 pyramidal cells and dentate gyrus granule cells [5].

At certain conditions defined by the membrane potential and by transmembrane concentration gradients of the transported ions, glutamate transporters may reverse transport and release glutamate. If and when the reverse occurs depends on glutamate transport stoichiometry (GTS), which ultimately affects neuronal survival. The GTS of glutamate transporters EAAT3, GLT-1 and GLAST has been determined previously as 3Na^+^: 1Glu^-^: 1H^+^: (-1)K^+^ per transport cycle [6–8]. All these published studies have been performed in the absence of chloride to avoid contaminating the measurement of the transporter current by the uncoupled chloride current, which is transmitted via an anionic channel associated with glutamate transporters [9,10]. However, the possibility of GTS variation with changes in glutamate concentration has never been explored.

There are four variants of GLT-1. Alternative splicing at 3’-ends of GLT-1 message leads to two splice variants: GLT-1a and GLT-1b, also known as GLT-1v, with different C-termini that are expressed in rat liver [11] and brain [12–15]. GLT-1b interacts with the synaptic PDZ domain protein PICK1 [16]. The third carboxyl-terminal splice-variant is GLT-1c that is found within the retina and in the synaptic terminals of both rod- and cone-photoreceptors in both humans and rats [17]. The fourth splice variant is an exon 9-skipping form of GLT-1a that is expressed primarily in populations of white matter astrocytes [18]. Remarkably, GLT-1a protein has been also found expressed in excitatory axon terminals in the CA3 and CA1 fields of the rat hippocampus [14,19,20] and elsewhere [21] where extracellular glutamate concentration varies dramatically during glutamatergic neurotransmission.

Extracellular glutamate concentrations in the synaptic cleft of glutamatergic synapses vary by five orders of magnitude from the resting 25 nM [22] to above 1 mM during normal excitatory transmission [23–25] and even higher during burst activity [26]. Such enormous variations in synaptic cleft glutamate concentration may affect GTS of synaptically and perisynaptically expressed GLT-1a during excitatory transmission. Previously we found that in primary neuronal cultures and GLT-1a transfected COS-7 cells, GLT-1 coupled transporter current exhibits a statistically significant decrease while [^3^H]-L-glutamate uptake monotonically increases when extracellular glutamate concentrations rises from 100 to 300 μM [27]. This change in the current/flux ratio implies GTS variation or in other words “slippage” of the transport coupling in this particular glutamate concentration range. Similar “slippages” in transport stoichiometry have been observed in other transporters including the bacterial lactose transporter, LacY [28], the ClC-ec1 H^+^-Cl^-^ exchange transporter [29], the dopamine transporter [30], the norepinephrine transporter [31], the serotonin transporter [32–34], and the Na^+^/Ca^2+^ exchanger [35].

Notably, the transport rate of the human homolog of rodent GLAST, EAAT1, at 300 μM glutamate had been found smaller relative to its rate at both 200 μM and 1000 μM (see Fig 5B in [36]), which is comparable to what we found for the GLT-1 coupled current [27]. However, the decrease in EAAT1 was not statistically significant due to the large errors of the means. Interestingly, in the following Fig 8D in [36], the authors presented the concentration dependence of the time constant for activation of L-glutamate currents, but without the 300 μM datum point. Therefore, our work clarifies the transport properties of GLT-1 specifically near 300 μM glutamate.

In the GTS studies of EAAT3, the coupled current was measured at 5, 10, 30, 100 and 300 μM, while the EAAT3 reversal potential was determined at the same concentrations, but not at the “critical” 300 μM. In addition, the data potentially allowed authors to determine GTS at three different concentration ranges: (5–10), (10–30), and (30–100) μM glutamate (see Figs 2F, 3A and 4A in [6]), by measuring ratios of the changes in the transporter reversal potential to the changes in the Nernst potential for glutamate in each of these ranges. The authors may have found that the slope in the lower concentration range (5–10 μM) was larger than in the middle (10–30 μM) range, as is noticeable in Figs 3A and 4A. However, the authors determined the EAAT3 stoichiometric ratio only across the entire glutamate concentration range, 5–100 μM. The reason is likely to be due to relatively large errors of the means of EAAT3 reversal potential measurements at individual glutamate concentrations, which would lead to large errors in the determination of the slopes and the stoichiometric ratios in these three concentration ranges. Interestingly, the measurement of the transporter reversal potential at the critical 300 μM glutamate was not communicated in this paper. It is possible that the reversal potential at 300 μM did not lie along the single fitting line with other the measurements in Fig 3A, and therefore would tend to indicate a change in the slope and in the stoichiometry, which would contradict the constant transport stoichiometry paradigm. This is another reason to measure GTS in at least two different glutamate concentration ranges around 300 μM.

GLT-1 stoichiometry has been previously determined, but also at a single glutamate concentration range, 100–300 μM [7]. Likewise, GLAST stoichiometry has been measured over a single range, 200–1000 μM [8]. Obviously to elucidate whether GTS of a transporter varies, one needs to know at least two different values of GTS measured in two different glutamate concentration ranges, which has never been done before.

Our previous report on the “slippage” of the transport coupling was conducted in the presence of Cl^-^ ions. Here we wanted to definitively ascertain the complete GTS across all relevant glutamate concentrations, and to do this we were compelled to omit Cl^-^. We evaluated GLT-1 transport stoichiometry at two different glutamate concentration ranges, in the absence of Cl^-^, which required measurements of GLT-1 reversal potential at a minimum of four different glutamate concentrations: two below and two above 200 μM. To clarify whether, at high glutamate concentration, GLT-1 operates in a channel-like mode, when GLT-1 thermodynamic parameter *Q*_*10*_ might diminish closer to 1, we measured the glutamate concentration dependence of the *Q*_*10*_ of the GLT-1 coupled transporter current. Our data show that in the absence of chloride, GLT-1 GTS remains constant over a wide 19–1,200 μM glutamate concentration range and over the same range shows a value of *Q*_*10*_ = 2.4. Surprisingly, the previously observed evidence for slippage of the GLT-1 coupled current was found to be dependent on the presence of chloride.

## Materials and Methods

### Assessment of GLT-1 transport stoichiometry

Here we assigned the reversal potential of GLT-1 as E_rev_, GLT-1 coupled current as *i*, and membrane potential as ϕ. In the linear approximation we can write

$$i=g(\phi -E_{rev})$$ (1)

where *g* is apparent stoichiometric (not-anionic) conductance of GLT-1 transporter.

At the reversal potential, *ϕ* = *E*_*rev*_, the current is zero and there is no change in total energy of the system per transport cycle. In this case, the change of the total energy alteration per cycle is equal to sum of changes in electrical and thermodynamic energies of transported ions and molecules, i.e. Σ(*n*_*j*_ *z*_*j*_)*eϕ* + Σ(*n*_*j*_ *U*_*j*_ / *N*_*A*_) = 0. Where, *n*_*j*_ – number of ions type “*j*” transported per cycle, *z*_*j*_−*j*-ion valence, *e*–elementary positive charge (1.6 × 10^−19^ coulombs), *U*_*j*_−transmembrane thermodynamic potential of *j*-ions that is equal to RT *ln* [(intracellular *j*-ion concentration) / (extracellular *j*-ion concentration)], and *N*_*A*_−the Avogadro constant (6.02 × 10^23^ mole^−1^). If all transported species are ions with non-zero charge, which is the case for GLT-1, the equilibrium at *ϕ* = *E*_*rev*_ can be rewritten in the following form:

$$\Sigma \left(n_{j}z_{j}\right)eE_{rev}–\Sigma \left(n_{j}z_{j}eE_{j}\right)=0$$ (2)

where, *E*_*j*_ = –*U*_*j*_ / (*z*_*j*_ *F*)–is Nernst equilibrium potential of “j”-ion.

Note that *U*_*j*_ = −*z*_*j*_ *FE*_*j*_.

From Eq 2 we find that

$$E_{rev}=\Sigma \left(n_{j}z_{j}E_{j}\right)/\Sigma \left(n_{j}z_{j}\right)$$ (3)

Thus, *E*_*rev*_ for a glutamate transporter can be calculated as:

$$E_{rev}=\left(n_{Na}E_{Na}+n_{H}E_{H}+n_{K}E_{K}-n_{Glu}E_{Glu}\right)/\left(n_{Na}+n_{H}+n_{K}-n_{Glu}\right)$$ (4)

Here we have *z*_*Na*_ = *z*_*H*_ = *z*_*K*_ = +1, *z*_*Glu*_ = −1. For example, in case of generally accepted GTS we have *n*_*Na*_ = 3, *n*_*H*_ = 1, *n*_*Glu*_ = 1, *n*_*K*_ = −1, and *n*_*e*_ = (*n*_*Na*_ + *n*_*H*_ + *n*_*K*_ − *n*_*Glu*_) = +2.

If glutamate is transported in neutral form, as a zwitterion, and free protons are not transported at all, then E_rev_ will depend on concentration of neutral glutamate ([Glu^0^]) but not on pH directly. However, pH will affect [Glu^0^]. In this case we have: n_H_ = 0, z_Glu_ = 0, and the E_Glu0_ is indeterminate. Therefore, in Eq 3 we need to replace E_Glu0_ with U_Glu0_.

$$E_{rev}=\left\{n_{Na}E_{Na}+n_{K}E_{K}-n_{Glu0}\left((RT/F)ln\left({\left[Glu^{0}\right]}_{i}/{\left[Glu^{0}\right]}_{e}\right)\right)\right\}/\left(n_{Na}+n_{K}\right)$$ (5)

For further consideration, we will take the generally accepted view that only the ionic form of glutamate is transported by GLT-1. When extracellular concentration of transported ion changes, E_rev_ changes too, and its shift can be calculated. For example, if we change the sodium concentration in Eq 4 then:

$$\Delta E_{rev,Na}=n_{Na}\Delta E_{Na}/\left(n_{Na}+n_{H}+n_{K}-n_{Glu}\right)$$ (6)

Therefore,

$$n_{Na}/\left(n_{Na}+n_{H}+n_{K}-n_{Glu}\right)\equiv n_{Na}/n_{e}=\Delta E_{rev,Na}/\Delta E_{Na}\equiv \Psi_{Na}$$ (7)

We use Ψ_Na_ as an abbreviation for the experimentally determined ratio of the shift of GLT-1 reversal potential to the shift of sodium Nernst potential. Remarkably, it is equal to the ratio of the number of the transported ions per one transported positive elementary charge.

Similar relations can be determined for the all remaining 3 ions:

$$n_{H}/\left(n_{Na}+n_{H}+n_{K}-n_{Glu}\right)\equiv n_{H}/n_{e}=\Delta E_{rev,H}/\Delta E_{H}\equiv \Psi_{H}$$ (8)

$$n_{K}/\left(n_{Na}+n_{H}+n_{K}-n_{Glu}\right)\equiv n_{K}/n_{e}=\Delta E_{rev,K}/\Delta E_{K}\equiv \Psi_{K}$$ (9)

$$n_{Glu}/\left(n_{Na}+n_{H}+n_{K}-n_{Glu}\right)\equiv n_{Glu}/n_{e}=-\Delta E_{rev,Glu}/\Delta E_{Glu}\equiv \Psi_{Glu}$$ (10)

Thus, the number of transported sodium ions per one transported glutamate is equal to:

$$n_{Na}/n_{Glu}=\left(n_{Na}/n_{e}\right)/\left(n_{Glu}/n_{e}\right)=\Psi_{Na}/\Psi_{Glu}$$ (11)

Similarly, we can determine the ratios for other ions:

$$n_{H}/n_{Glu}=\Psi_{H}/\Psi_{Glu}$$ (12)

$$n_{K}/n_{Glu}=\Psi_{K}/\Psi_{Glu}$$ (13)

Interestingly, since n_Na_ + n_H_ + n_K_—n_Glu_ = n_e_, then the experimental values should be in agreement with the following theoretical equation:

$$n_{Na}/n_{e}+n_{H}/n_{e}+n_{K}/n_{e}-n_{Glu}/n_{e}=\Psi_{Na}+\Psi_{H}+\Psi_{K}–\Psi_{Glu}=1$$ (14)

Therefore, generally excepted stoichiometric ratios give us the anticipated result: 1.5 + 0.5–0.5–0.5 = 1. Our data gave us also the same result: 1.6 + 0.5–0.6–0.5 = 1.0. Thus, by measuring shifts of E_rev_ of the transport and knowing changes of ionic concentrations associated with them we can unambiguously determine the complete stoichiometry of the transporter.

The stoichiometric ratios calculated from E_rev_ shifts published for different glutamate transporters and those found for GLT-1 in this study are presented in Table 1.

**Table 1:** Stoichiometric transport ratios of glutamate transporters in the absence of chloride that have been reported previously and found here.

Transporter	[Glu]_e_ (μM) range	Major anion	Temperature (°C)	n_Na_/n_e_	n_Glu_/n_e_	n_H_/n_e_	n_K_/n_e_	Source
**EAAT3**	**5–100**	Gluconate	Unspecified	1.70	0.54	0.42	-0.53	Zerangue et al., 1995
**GLT-1**	**100–300**	Gluconate	Unspecified	1.52	0.50	0.52	-0.39	Levy et al., 1998
**GLAST**	**200–1000**	Gluconate	Unspecified	1.37	0.43	0.40	-0.40	Owe et al., 2006
**GLT-1**	**19–75**	D-gluconate	37	1.56 ±0.23	0.50 ±0.02	0.48 ±0.06	-0.60 ±0.05	This study
**GLT-1**	**300–1200**	D-gluconate	37	1.48 ±0.13	0.52 ±0.04	0.48 ±0.03	-0.58 ±0.06	This study
**GLT-1**	**19–75**	D-gluconate	26	1.44 ±0.20	0.48 ±0.02	0.37 ±0.07	-0.60 ±0.12	This study
**GLT-1**	**300–1200**	D-gluconate	26	1.33 ±0.20	0.46 ±0.05	0.43 ±0.06	-0.65 ±0.10	This study

[Glu]_e,1_ and [Glu]_e,2_ are two glutamate concentrations which were used to determine number of glutamate molecules transported per one elementary charge (e^+^) in an appropriate study. Data are shown as mean ± s.e.m.

### Neuronal cultures

The Children’s Hospital Institutional Animal Care and Use Committee (IACUC) approved this study. Neuronal cultures were prepared from embryonic day 16 Sprague Dawley rat fetuses as described previously [12,37]. Contamination by astrocytes was determined by immunochemical labeling with anti-glial fibrillary antibody and was found to be < 0.2% of total cells. Cultured neurons were used in electrophysiological experiments between 14 to 28 days *in vitro*.

### Transient transfection of COS-7 cells by EGFP and GLT-1a cDNAs

For GLT-1a stoichiometric measurements we used COS-7 cells, which is a African green monkey kidney fibroblast-like cell line [38] suitable for transfection by vectors requiring expression of SV40 T antigen (ATCC, catalog No. CRL-1651). 0.3 million wild type COS-7 cells were plated per 35 mm dishes in VP-SFM (cat. # 11681–020, Invitrogen, Carlsbad, CA) with 4 mM L-glutamine. In 24 hours cell medium in each dish was replaced with 2.4 ml Opti-MEM (cat. # 31985–062, Invitrogen) containing 4 μg GLT-1a and 1 μg EGFP cDNAs [12] and 12 μl Lipofectamine 2000 (cat.# 11668–019, Invitrogen) for 5 hours. Then the transfection medium was replaced with VP-SFM containing 0.1 mM L-glutamine. After 24–72 hour the transfected COS-7 cells with maximum EGFP fluorescence were selected for the patch-clamp experiments using fluorescent DIAPHOT 200 inverted microscope (Nikon, Japan). In separate experiments, COS-7 cells were transfected with EGFP only for control patch-clamp experiments.

### Electrophysiology

There are two different currents generated by GLT-1 that are activated by glutamate. The current of interest is stoichiometrically coupled with glutamate transport, but another current is uncoupled with the glutamate transport and generated by chloride ions passing through anionic channel inside the transporter [3]. In the experimental solutions where chloride was the major anion, we excluded the uncoupled anionic current by nullifying the driving force for chloride. This was accomplished by equalizing the Nernst equilibrium potential for chloride (E_Cl_) to the holding membrane potential (HP) of −70 mV. In these conditions the current generated by GLT-1 was only the coupled glutamate transporter current (*i*_*Glu*_).

Electrophysiological experiments were performed in the whole cell configuration with WPC-100 amplifier (E.S.F., Germany), pClamp 10 software (Molecular Probes, USA), and MP-225 micromanipulator (Sutter Instrument, USA). Only cell with more than 5 GOm seal resistance at cell-attached configuration were used in the experiments to minimize leak current and improve signal to noise ratio for the small GLT-1 current. Temperature of the chamber (Cat. # TC-344B, Warner Instruments) was adjusted to 37°C or to 26°C as described below. Extracellular solution exchange was performed with computer controlled fast solution application system (Cat. # PC-1, Bioscience Tools, USA) with 8-channel heated micromanifold MPRE8 (Cell MicroControls, USA) that allowed to change solution around a studied cell within 300 ms and adjust solution temperature with 0.1°C accuracy. The temperature of the solution coming from the manifold was also adjusted to 37°C or to 26°C, accordingly to appropriate experimental protocols. The control solution was always applied before and after each experimental solution to minimize effect of the base line drift and improve accuracy of GLT-1 reversal potential (E_rev_) measurements. Several current records obtained during subsequent applications of the same set of solutions were later averaged to improve signal to noise ratio.

Membrane conductance was determined as an average of two conductances at positive and negative slopes of the voltage ramp in -70 to -30 mV regions. The changes in the membrane capacitance were determined from the differences of the currents at positive and negative slopes of the voltage ramp according to equation: ΔC = ΔI/Δ(dV_m_/dt).

### Solutions

Artificial cerebrospinal fluid (ACSF) contained (in mM): 160 NaCl, 2.5 KCl, 2.5 CaCl_2_, 1.3 MgCl_2_, 10 D-glucose, 10 HEPES (pH 7.4, adjusted with N-methyl-D-glucamine). The intracellular solution contained (in mM): 1 NaCl, 110 KOH, 20 tetraethylammonium (TEA), 1 CaCl_2_, 5 MgCl_2_, 5 EGTA, 10 HEPES (pH 7.2 was adjusted with gluconic acid). Osmolality in all solutions was measured with (Wescor vapor pressure osmometer Model 5520, Logan, UT, USA) and adjusted to 330 mmol/kg with mannitol. With these solutions E_Cl_ = -70 mV that allows us to nullify GLT-1 uncoupled chloride current at HP = −70 mV. In addition, intracellular potassium was adjusted to obtain E_K_ = -70 mV, by replacement with N-methyl-D-glucamine to nullify all currents through the potassium channels to increase signal to noise ratio for GLT-1 couple current measurements. In experiments with cultured cerebral neurons, all ionotropic glutamate receptors were blocked with 10 μM (*RS*)-3-(2-carboxypiperazin-4-yl)-propyl-1-phosphonic acid (CPP), 100 μM 5,7-dichlorokynurenic acid, 10 μM 2,3-dioxo-6-nitro-1,2,3,4-tetrahydrobenzoquinoxaline-7-sulfonamide (NBQX), and 50 μM 4-(8-methyl-9*H*-1,3-dioxolo[4,5-h][2,3]benzodiazepin-5-yl)-benzenamine (GYKI 52466) [39]. Endogenous neuronal electrical activity was blocked with 0.3 μM TTX. To reduce GABA associated noise we added 25 μM bicuculline.

To measure shifts of GLT-1 E_rev_ we used GLT-1a transfected COS-7 cells that have no glutamate activated channels and high expression level of GLT-1a. In intra- and extracellular solutions all chloride was substituted with D-gluconate, an impermeable anion. Chloride ions are required for current transduction at the solution to silver-chloride pellet interface. Durations of all our experimental protocols were less than 100 seconds, and the average GLT-1 current was always below 1000 pA. Therefore, total required transmitted charge was less than 10^−7^ C, corresponding to 1.04 pmol of Cl^-^ as the charge carrier, which in 10 μl of the pipette solution translates into approximately 100 nM for the pipette Cl^-^ concentration. Such a miniscule amount of chloride in the pipette solution might come either from the traces of chloride in the salts used to make up the solution or from chloride dissociation from the silver-chloride pellet during the several minutes before the start of an experiment. The solution for the electrical connection with the reference electrode was ACSF with high chloride concentration. The chloride-free extracellular solution contained (in mM): 102 Na D-gluconate, 44 K D-gluconate, 10 D-glucose, 15 HEPES, pH 7.4 adjusted with N-methyl-D-gluconate. Concentrations of free calcium and magnesium were adjusted to 1 mM and 2 mM, respectively, with Ca(D-gluconate)_2_ and Mg(D-gluconate)_2_, and confirmed by measurements with ion-selective electrodes. The intracellular solution in these experiments contained (in mM): 20 Na D-gluconate, 10 Na L-glutamate, 100 K D-gluconate, 20 TEA, 5 EGTA, 20 HEPES, pH 7.0 adjusted with D-gluconic acid. Concentrations of free calcium and magnesium were adjusted to 100 nM and 4 mM with Ca(D-gluconate)_2_ and Mg(D-gluconate)_2_, respectively, and measured with ion-selective electrodes. Osmolality in all solutions was adjusted to 330 mmol/kg with mannitol. To determine the ion specific shift of the GLT-1 E_rev_, the following ionic concentrations were changed in separate experiments: 1) [Na^+^]_e_ from 102 to 51 mM, 2) [K^+^]_e_ from 42.5 to 10.6 mM, and 3) [H^+^]_e_ from 40 to 10 nM (that corresponds to a pH shift from 7.4 to 8.0) in the presence of 37.5 or 600 μM glutamate. Since the inhibition of GLT-1 by DHK is competitive, we have used higher DHK concentrations at higher glutamate concentrations to determine E_rev_ of DHK sensitive current more accurately that is described in details in appropriated sections below.

### Statistics and fitting of the data

Experimental data in the figures and in the text are presented as mean ± s.e.m. Two-tailed t-test was used to determine statistical significance of the effects.

For comparison of the ratios of GLT-1 current in the presence and the absence of chloride to appropriate flux data in the presence of chloride obtained previously [27], we have normalized the data at 100 μM glutamate to corresponding values at 300 μM glutamate. In curve fittings, we used Origin (version 6; Microcal Software Inc.).

## Results

### How many Na^+^ ions does GLT-1 transport per one positive elementary charge (e^+^)?

**Fig 1:**
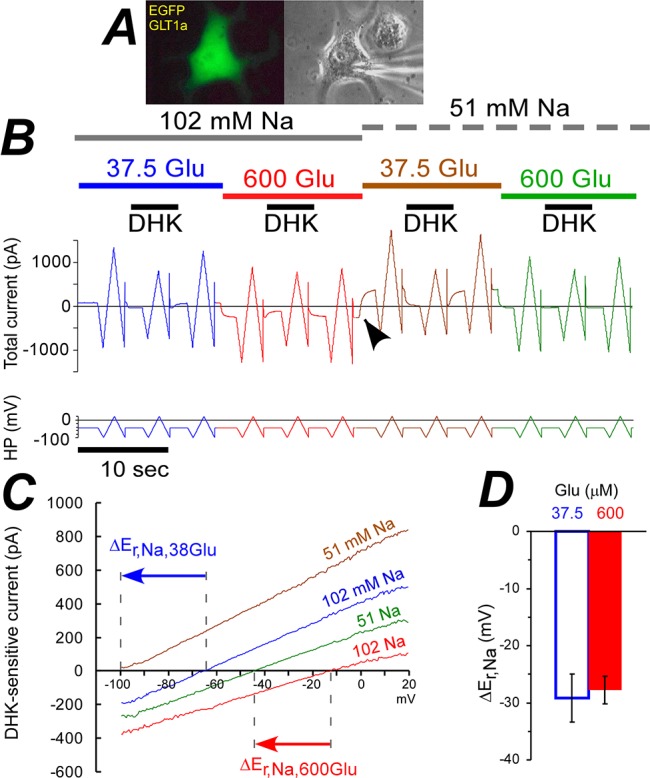
In the absence of chloride, two-fold decrease in [Na^+^]_e_ shifted GLT-1a E_rev_ by −28 mV at both low and high glutamate concentrations. **(A)** Fluorescent (left) and phase-contrast (right) images of COS-7 cell expressing both EGFP and GLT-1a. **(B)** Representative data show total membrane current, the time courses of [Na^+^]_e_, [Glu^-^]_e_, and [DHK]_e_ at the top, and HP voltage ramps (–100 to +20 mV) at the bottom. Arrow head points to simultaneous decrease in [Glu^-^]_e_ and [Na^+^]_e_, which affects the current. **(C)** I-V curves of DHK sensitive currents obtained form **(B)** as a result of subtractions of averages of 2 I-V curves in presence of DHK from averages of 4 I-V curves in the absence of DHK at corresponding [Glu^-^]_e_ and [Na^+^]_e_. Arrows show amplitudes and directions of E_rev_ shifts associated with decrease in [Na^+^]_e_. **(D)** Average E_rev_ shifts at [Glu^-^]_e_ geometric means of 37.5 and 600 μM are approximately equal when [Na^+^]_e_ was decreased from 102 to 51 mM. Data here and in all following bar graphs are shown as mean and SEM.

Previously we observed decrease in n_e_/n_Glu_ at high glutamate concentrations measured in the presence of chloride [27]. Such a change might be due to an increase in n_Glu_, but also might be due to changes in n_Na_, n_H_, or n_K_, which all affect n_e_. To determine exact GLT-1 GTS at both low and high glutamate concentrations we used COS-7 cells transiently transfected with both GLT-1a and enhanced green fluorescent protein (EGFP) (Fig 1A). All stoichiometric experiments were performed in chloride-free solutions to avoid possible contamination of the measured coupled transporter current by the uncoupled anionic current.

The calculations of GLT-1 stoichiometric ratios from the shifts of its reversal potential are described in the Methods section. E_rev_ was determined as the zero-current potential of the current blocked by 600 or 700 μM DHK at 37.5 or 600 μM glutamate, respectively (Fig 1B and 1C). When [Na^+^]_e_ was decreased 2 times (replacement by non-permeable N-methyl-D-gluconate), E_rev_ shifted by approximately 28 mV in the negative direction at both 37.5 and 600 μM glutamate concentrations (Fig 1D). Thus, n_Na_/n_e_ ratio is the same at both low and high glutamate concentrations; n_Na_/n_e_ = ΔE_rev_/ΔE_Na_ ≈ (-28 mV) / (RT/F ln(51/102)) ≈ 1.5.

### GLT-1 transports one Glu^-^ per two e^+^ at low and high [Glu]_e_

**Fig 2:**
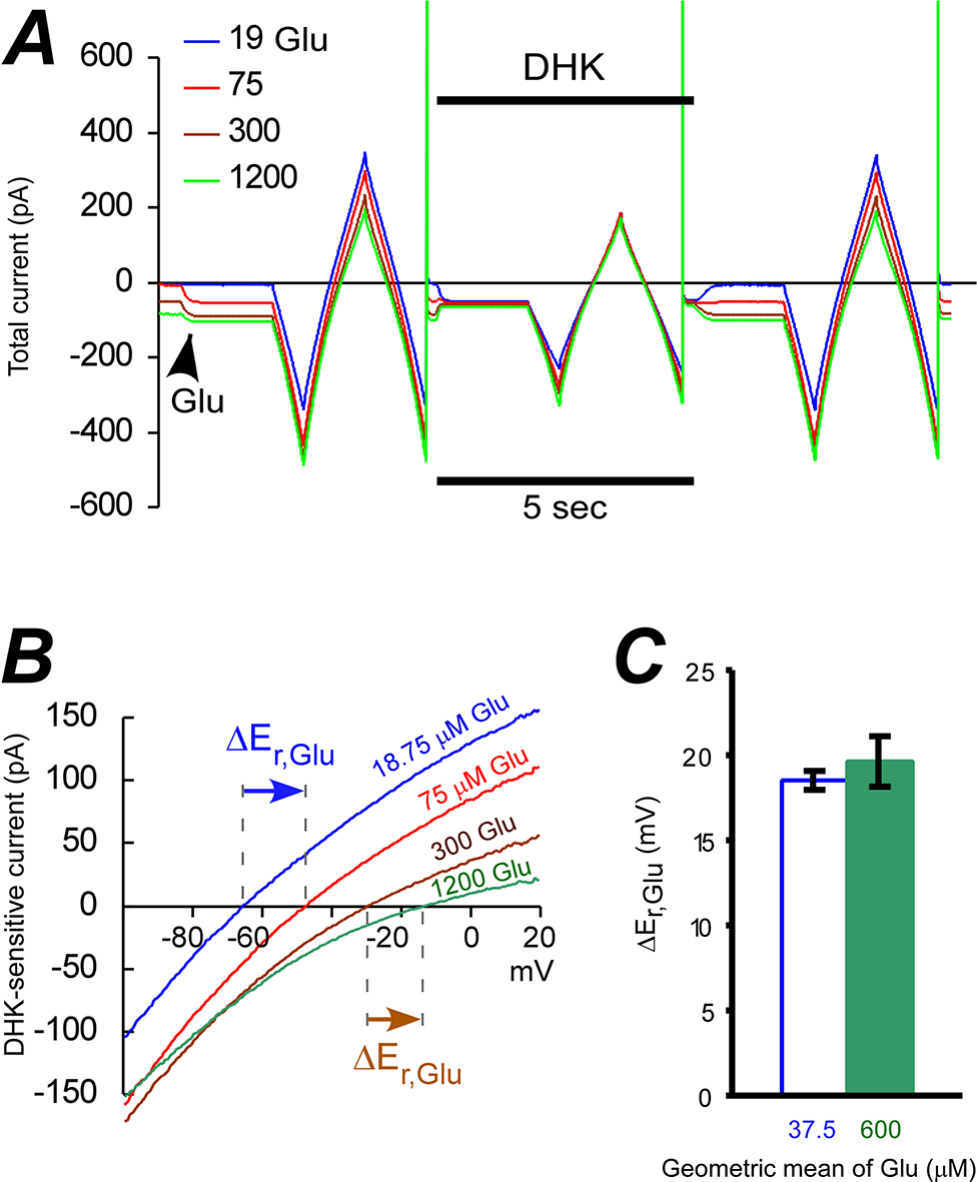
Four-fold increase in [Glu^-^]_e_ shifted GLT-1a E_rev_ by +19 mV at both low and high glutamate concentrations. **(A)** Representative experiment shows responses of the total current to the voltage ramp (–100 to +20 mV) at different [Glu^-^]_e_ in the absence and the presence of DHK. Arrow head shows onset of next glutamate concentration application. **(B)** I-V curves of DHK sensitive currents obtained form **(A)**. Arrows show E_rev_ shifts. **(C)** Average GLT-1 E_rev_ shifts at [Glu^-^]_e_ geometric means of 37.5 μM and 600 μM are approximately equal when [Glu^-^]_e_ was increased from 18.75 to 75, or from 300 to 1200 μM, respectively.

The n_Glu_/n_e_ ratios were determined by measuring the ΔE_rev_ of the DHK sensitive current when [Glu^-^]_e_ was increased from 18.75 to 75, to 300, and to 1200 μM. Due to the competitive nature of DHK inhibition, we used increasing concentrations of [DHK]_e_ (Fig 2A). The first four fold increase in [Glu^-^]_e_ from 18.75 to 75 μM, corresponding to the geometric mean of 37.5 μM, led to approximately 19 mV shift of the E_rev_ of DHK-sensitive current in positive direction (Fig 2B). Similarly, the last four fold increase in [Glu^-^]_e_, with its geometric mean equal to 600 μM, led to the same ΔE_rev_ (Fig 2C). Therefore, n_Glu_/n_e_ = -ΔE_rev_/ΔE_Glu_ ≈ (+19 mV) / (RT/F ln(4)) ≈ 0.5 in both examined glutamate concentration ranges. Surprisingly, these results challenge our own current/flux studies where the ratio at high glutamate concentration was approximately two times lower than at low concentration. However, our previous current/flux studies were performed in the presence of chloride [27], which we will address below.

### GLT-1 cycle time is 52 ms in the absence of chloride

The experiments shown in Fig 2 allowed us to determine the membrane capacitance at each pair of positive and negative holding potential (HP) ramps as described in the Methods section. We found that at 37°C, −50 mV and 1200 μM glutamate, the ratio of the DHK-sensitive GLT-1 current to DHK-sensitive capacitance is equal to 4.6±0.4 (A/F = V/s). Therefore, at −50 mV the ratio of the current to GLT-1-specific capacitive charge is equal to (4.6 V s^-1^) / (50 mV) = 92±8 s^-1^, which is close to the value of 70 s^-1^ found at – 80 mV and 1000 μM glutamate for human EAAT2 [40]. This number (92±8 s^-1^) would correspond to the charge transfer rate if the GLT-1 capacitive charge would be equal to an elementary charge and sensed the whole membrane potential. However, assuming that the product of the valence of the capacitive charge and the apparent fraction of the field sensed by the charge (zδ) in GLT-1 is the same as in EAAT2 and equal to 0.41 [40], the number of elementary charges transported at −50 mV per sec by GLT-1 is 38±3 s^-1^, which is relatively close to the value of 29 s^-1^ shown for EAAT2 at −80 mV [40]. Therefore, when GLT-1 transports 2 elementary charges per cycle (2 = +3_Na_− 1_Glu_ + 1_H_ – 1_K_), its cycle time is equal to 52±4 ms, which corresponds to the turnover rate of 19±2 s^-1^.

### GLT-1 counter-transports one K^+^ ion per two e^+^ at low and high [Glu]_e_

**Fig 3:**
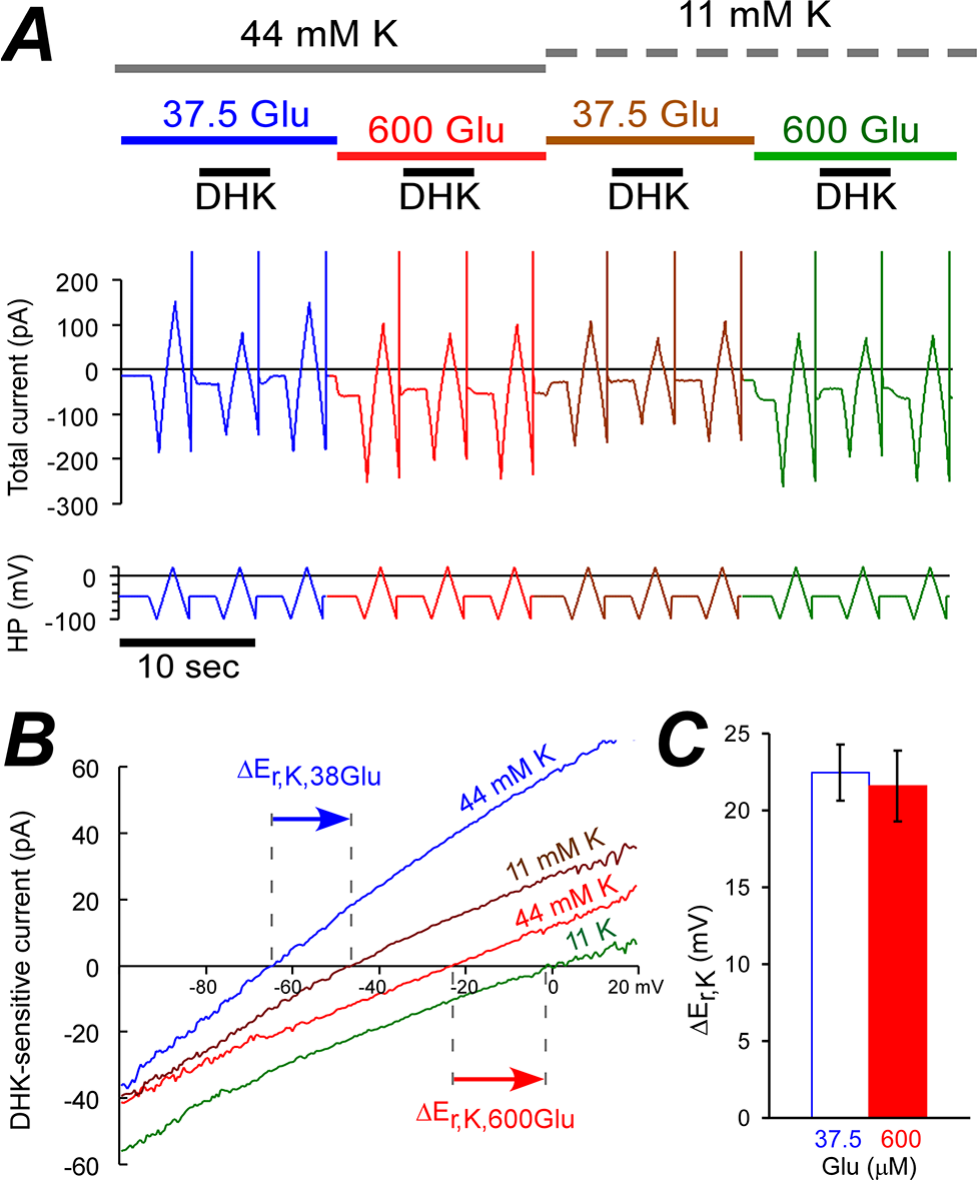
Four-fold decrease in [K^+^]_e_ shifted GLT-1a E_rev_ by +22 mV at both low and high glutamate concentrations. **(A)** Representative experiment shows the current with the identifying clues as in Fig 1B. **(B)** I-V curves of DHK sensitive currents obtained form **(A)**. Arrows show GLT-1a E_rev_ shifts. **(C)** Average GLT-1 E_rev_ shifts at 37.5 μM and 600 μM are approximately equal when [K^+^]_e_ was decreased from 44 to 11 mM.

These experiments were similar to those described in Fig 1, with different [K^+^]_e_ applied as shown in Fig 3A. We used 600 or 700 μM DHK at 37.5 or 600 μM glutamate, respectively (Fig 3A and 3B). When extracellular potassium concentration was decreased four times from 44 to 11 mM, E_rev_ of the DHK sensitive current shifted by 22.5±1.8 mV and by 21.6±2.3 mV in positive direction for 37.5 and 600 μM glutamate, respectively (Fig 3C). Therefore, n_K_/n_e_ ratio is essentially the same for both low and high glutamate concentrations. That is n_K_/n_e_ = ΔE_rev_/ΔE_K_ ≈ (+22 mV) / (RT/F ln(11/44)) ≈ -0.57.

### GLT-1 transports one proton per two e^+^ at low and high [Glu]_e_

**Fig 4:**
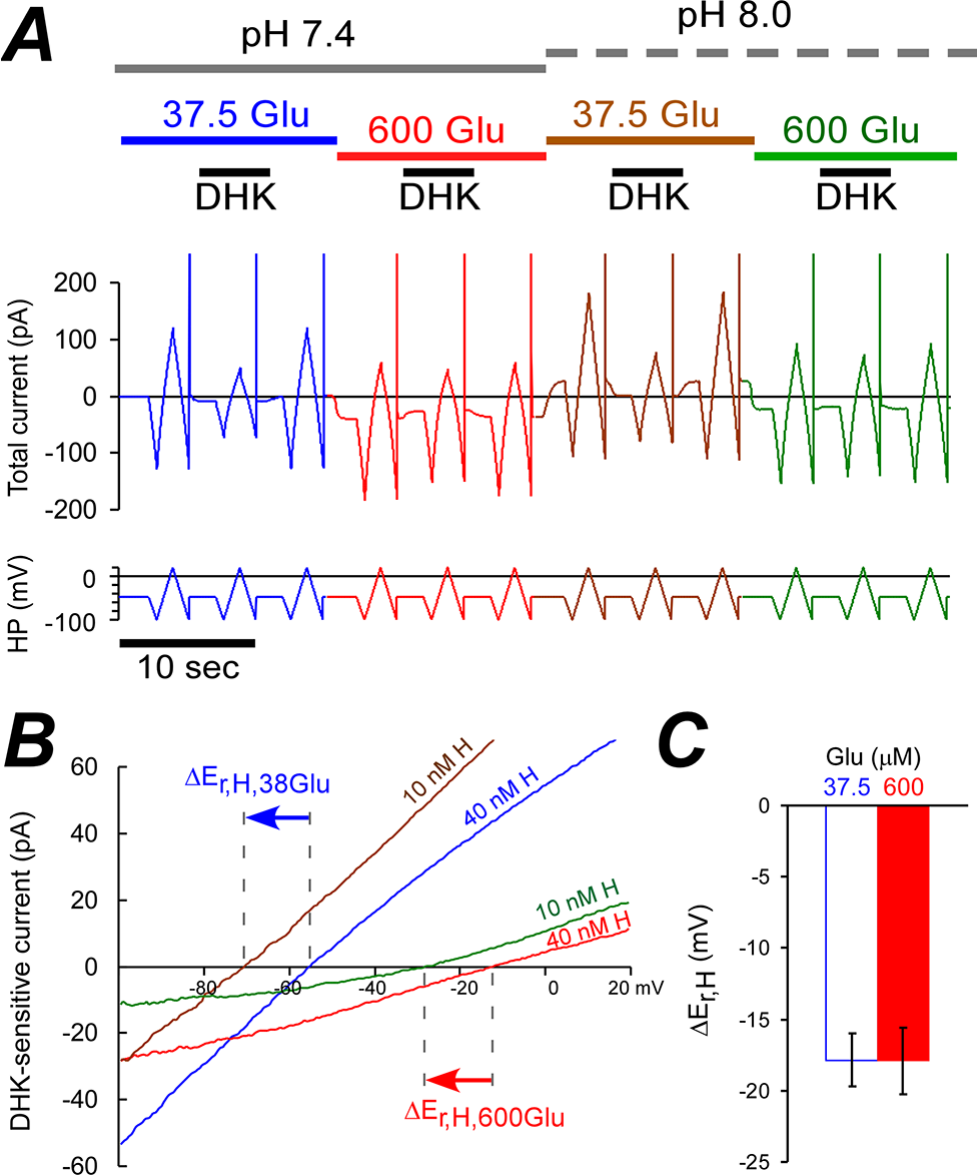
Four-fold decrease in [H^+^]_e_ shifted GLT-1a E_rev_ by −18 mV at both low and high glutamate concentrations. **(A)** Representative experiment shows the current as in Fig 1B. **(B)** I-V curves of DHK sensitive currents are obtained form **(A)**. Arrows show GLT-1a E_rev_ shifts. **(C)** Average GLT-1 E_rev_ shifts at 37.5 μM and 600 μM are approximately equal when [H^+^]_e_ was decreased from 40 to 10 nM.

We have determined the effect of the proton concentration on GLT-1 E_rev_ as shown in Fig 4A. We used 600 or 700 μM DHK at 37.5 or 600 μM glutamate, respectively (Fig 4A and 4B). When extracellular proton concentration was decreased four times from 40 to 10 nM (i.e. pH was increased from 7.4 to 8.0), E_rev_ of the DHK sensitive current shifted by 18±2 mV and by 18±1 mV in negative direction for 37.5 and 600 μM glutamate, respectively (Fig 4C). Therefore, n_H_/n_e_ ratio is the same for both low and high glutamate concentrations; i.e., n_H_/n_e_ = ΔE_rev_/ΔE_H_ = (-18 mV) / (RT/F ln(10/40)) = 0.48.

### In the absence of chloride GLT-1 GTS does not depend on temperature

**Fig 5:**
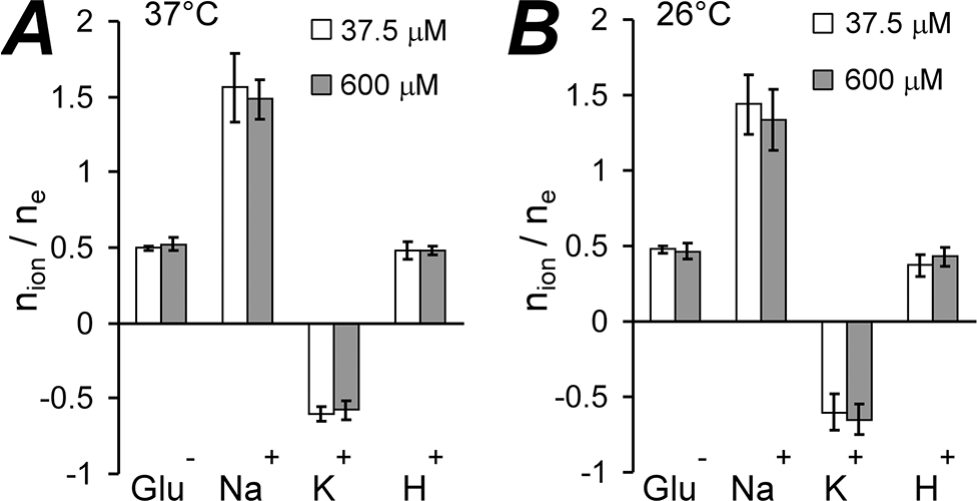
In the absence of chloride, GLT-1 glutamate transport stoichiometry remains constant at low and high glutamate concentrations, as well as at different temperatures. **(A)** GLT-1 transport stoichiometry at 37°C at low (open bars) and high (grey bars) glutamate concentrations. For pair of transported elementary charges GT1 co-transports approximately one glutamate, three sodium ions, and one proton, and counter-transports one potassium ion. **(B)** GLT-1 transport stoichiometry at 26°C at low (open bars) and high (grey bars) glutamate concentrations. The stoichiometry is the same as at 37°C and approximately has the following transport ratios: n_Glu-_/n_e+_ = ½, n_Na+_/n_e+_ = 3/2, n_K+_/n_e+_ = -½, and n_H+_/n_e+_ = ½.

So far the data imply that in the absence of chloride, the transport stoichiometry at 37°C remains the same at both 37.5 and 600 μM glutamate (Fig 5A). This is in agreement with the generally accepted point of view, but does not support our previous data [27] where we discovered slippage of GLT-1 current-flux coupling at 37°C with k_D_ = 0.17 mM glutamate and n_Hill_ = 3.2. Some investigators have not specified the temperature used in their studies of GTS (see Table 1), and therefore we conducted a similar set of stoichiometric experiments at 26°C. However, GLT-1 GTS still remained constant even at the lower temperature (Fig 5B).

### In the absence of chloride, *Q*_*10*_ for GLT-1 transport does not depend on [Glu]_e_

**Fig 6:**
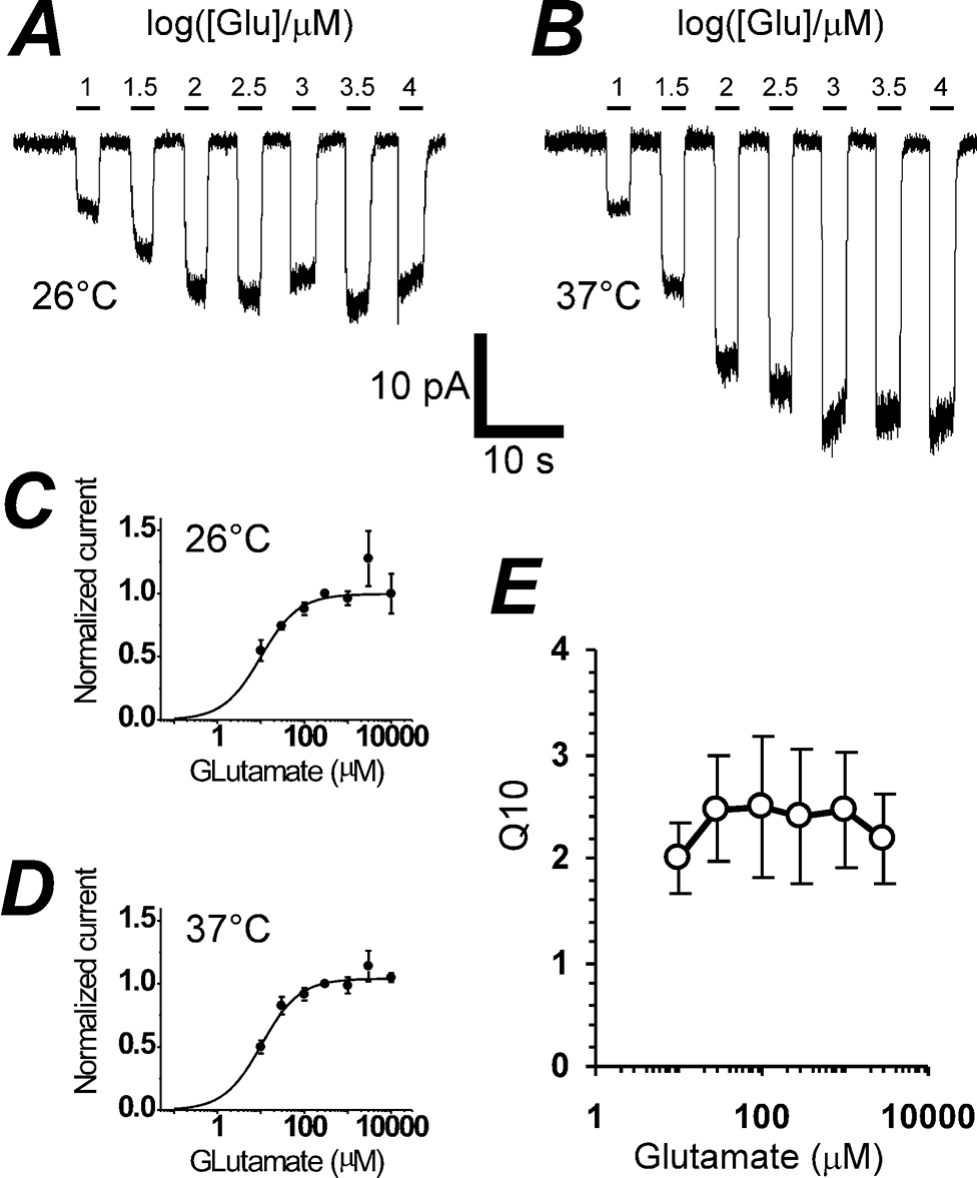
In the absence of chloride, *Q*_*10*_ of GLT-1a does not depend on glutamate concentration. **(A)** Representative experiment shows responses of GLT-1a stoichiometric current to wide range of glutamate concentrations (from 10 to 10^4^ μM) at 26°C. **(B)** Glutamate responses of the same cell as in **(A)** at 37°C. The scaling bars are the same for **(A)** and **(B)**. **(C)** and **(D)** show cumulative data for 26°C and 37°C with glutamate k_D,26°C_ = 9.9 ± 1.6 μM and k_D,37°C_ = 10.3 ± 1.5 μM, respectively. (*E*) Glutamate dose dependence of *Q*_*10*_ of GLT-1a. The average value of *Q*_*10*_ for glutamate concentrations in the range from 30 μM to 10 mM is 2.4 ± 0.1.

We measured *Q*_*10*_ for GLT-1 coupled current in 10 μM– 10 mM glutamate concentration range (Fig 6). The *Q*_*10*_ value remained constant (*Q*_*10*_ = 2.4±0.1) over the whole range, and does not comes close to the value for passive diffusion where *Q*_*10*_ ≈ 1.3. This is in agreement with constant transport stoichiometry, and does not provide evidence for slippage of the current-flux coupling, because slippage is usually associated with channel-like behavior of a transporter and a lower *Q*_*10*_ value.

### Removal of chloride eliminates slippage of transport coupling in cultured neurons

The major difference in the present stoichiometric experiments compared to our previous experiments was the absence of chloride, leading to the hypothesis that the substitution of gluconate for chloride in the present experiments eliminated the slippage phenomenon. To test this hypothesis, we determined the coupled GLT-1 current-glutamate concentration dependence as we have previously [27], but with chloride replaced with gluconate. Surprisingly, we found that the decrease in the coupled transporter current in the 100–300 μM glutamate concentration range in GLT-1a transfected COS-7 cells was NOT observed in the absence of chloride (Fig 6A–6D).

**Fig 7:**
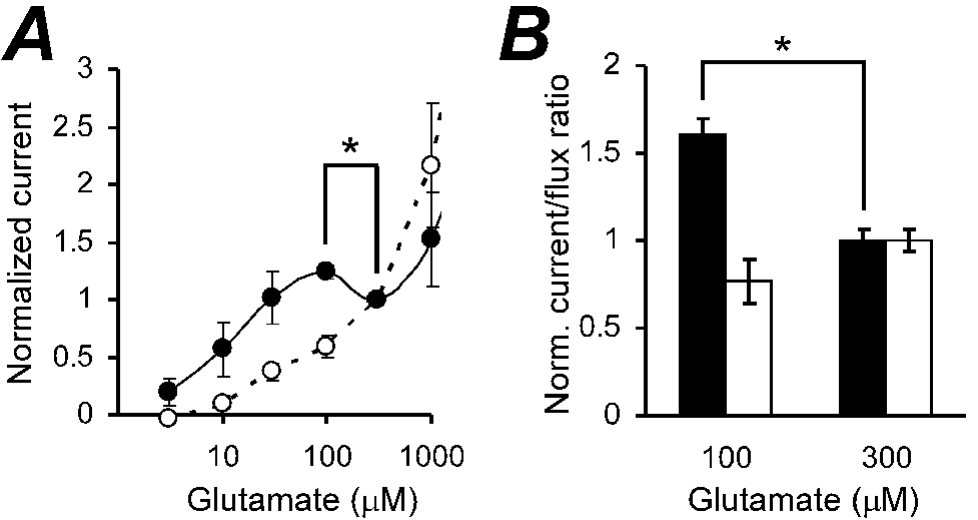
Replacement of intra- and extracellular chloride for D-gluconate eliminated statistically significant decreases in GLT-1 stoichiometric current and the current/flux ratio in 100–300 μM glutamate concentration range. **(A)** Normalized GLT-1 stoichiometric current glutamate concentration dependences in the presence chloride, E_Cl_ = HP = -70 mV, (solid circles), and in the absence of chloride (open circles). **(B)** Normalized current/flux ratios in the presence (black bars) and in the absence of chloride (white bars). Flux data are taken from [27]. *- corresponds to p < 0.05.

Our previous experiments were performed on primary neurons in culture [27]. The transporter current in cultured neurons significantly decreased in the 100–300 μM glutamate concentration range in the presence of chloride as has been described previously [27]. However, when we repeated similar experiments in the absence of chloride, we found that there was NO decrease in the transporter current in the same glutamate concentration range (Fig 7A). It should be noted that a non-saturating and GLT-1 non-specific current in the presence of chloride appears only at [Glu]_e_ > 300 μM, as it was shown in Figs 2D and 4C in [27]. Therefore, the increase in GLT-1 coupled current in neurons in the absence of chloride when [Glu]_e_ increases from 100 to 300 μM, and the decrease of GLT-1 current in the presence of chloride at the same concentrations should be considered as GLT-1 specific effect of chloride ions on the transporter (Fig 7A).

If we compare normalized ratios of the neuronal GLT-1 coupled current (in the presence and in the absence of chloride) to previously found values of [^3^H]-L-glutamate fluxes carried by neuronal GLT-1 at 100 and 300 μM [27], we find that the slippage (i.e. significant change in the current/flux ratio at 300 μM glutamate) occurs only in the presence of chloride (solid bars in Fig 7B, p < 0.05), but not in the absence of chloride (open bars).

## Discussion

### GLT-1 GTS is constant with substitution of gluconate for chloride

Analysis of GLT-1 glutamate transport thermodynamic equilibrium allowed us to derive Eqs (7–10, see the methods) showing that the number of a particular type of ion (atom or molecule) transported per elementary charge is equal to the ratio of the transporter reversal potential shift to the particular ion’s Nernst equilibrium potential shift. Our data indicate that in the absence of chloride, GLT-1 co-transports 1.5 Na^+^, 0.5 Glu^-^, 0.5 H^+^ and counter-transports 0.6 K^+^ per one transported elementary charge in both low (19–75 μM) and high (300–1200 μM) extracellular glutamate concentration ranges, and at both 26°C and 37°C temperatures (Figs 1–5). However, this is obviously inconsistent with the previously found slippage of transport coupling in GLT-1 at high glutamate concentrations in the presence of chloride [27], which we will discuss below.

The *Q*_*10*_ value of 2.4 remains constant in 30–10,000 μM glutamate concentration range (Fig 6), which is in agreement with the idea that GTS is constant and GLT-1 is not slipping into a channels-like mode when *Q*_*10*_ would diminish closer to 1. Found here *Q*_*10*_ value is close to the 2.9 value of *Q*_*10*_ for EAAT1 radiolabeled uptake [36]. This magnitude of *Q*_*10*_ is also in agreement with the temperature dependence of decay rate of synaptically activated transporter-mediated current in hippocampal astrocytes [26].

### The apparent slippage of GLT-1 transport coupling is not an artifact

The inconsistency between the constant GLT-1 stoichiometry found here and the variable stoichiometry in the presence of chloride [27] (Fig 7) might be an artifact in the current-flux experiments. However, we already evaluated plausible artifacts in previously reported experiments [27]. Briefly, the transport “slippage” is not due to accumulation of intracellular submembrane glutamate that could attenuate the expected increase in transporter driving force due to glutamate diffusion limitation in the submembrane space [40], similar to the one found for ATP [41]. “Slippage” was also observed at 10 mM intracellular glutamate, when the relative change in intracellular submembrane glutamate concentration would be negligible. The slippage is also not due to a self-blocking effect of glutamate on its own influx as had been shown for GABA_A_ receptors [42] because this would also decrease glutamate uptake, which did not occur. Likewise, it is not due to poor voltage camp that could lead to non-zero driving force for uncoupled chloride current [9] even when HP = E_Cl_, because uncoupled current was found to be very small in EAAT2 [40,43], and in addition the “slippage” phenomenon was also observed in GLT-1a transfected COS-7 cells, which are geometrically compact and therefore have reliable spatial voltage clamp [27]. The COS-7 experiments also exclude any possibility of GluRs contribution because GluRs are not expressed in these cells.

### Dependence of GLT-1 GTS on glutamate concentration depends on chloride

Previously [6–8], and here, GTS parameters have been determined in the absence of chloride to avoid the confound of a contribution from the uncoupled anionic transporter current to the measurements of the coupled current reversal potential. Yet, all previously published studies determined GTS only in a specified glutamate concentration range, and the possibility of GTS variation was not considered. Since we have found evidence for the slippage in GLT-1 transport coupling at high glutamate concentrations [27], it was obligatory to clarify if the complete transport stoichiometry is different in two [Glu]_e_ concentration ranges, specifically, below and above the apparent slippage phenomenon occurs. Unexpectedly, we found that GLT-1 GTS is the same in both low (19–75) and high (300–1200 μM) glutamate concentration ranges, as well as at both 37°C and 26°C temperatures (Fig 5). Specifically, when chloride is substituted by impermeable D-gluconate, GLT-1 GTS is 3Na^+^: 1Glu^-^: 1H^+^: (-1)K^+^, which is in agreement with the original reports [6–8].

To clarify the nature of the inconsistency between the slippage in GLT-1 transport coupling [27] and constant GTS found here, we assessed the coupled transporter current as a function of glutamate concentration in the identical experiments as before [27], but in the absence of chloride. In this case, we observed no reduction of the coupled transporter current in 100–300 μM glutamate concentration range in both GLT-1a transfected COS-7 cells (Fig 6) and neurons (Fig 7). These data imply that both GLT-1 transport coupling and GTS are constant in the absence of chloride ions, but vary in the presence of chloride and high glutamate concentrations.

### How to interpret effect of chloride on GLT-1 GTS?

There are at least two obvious possibilities that could potentially explain why GLT-1 transport coupling slippage occurs at high glutamate concentrations in the presence of chloride [27] and does not occur when chloride is absent (Figs 6 and 7). The first option is that chloride, but not D-gluconate, is required for a second glutamate transport with apparent k_D_ = 0.17 mM glutamate and glutamate binding cooperativity corresponding to n_Hill_ = 3.2 [27]. Noticeably, the latter matches n_Hill_ = 3 for the anionic uncoupled current of EAAT4, which occurs only at high transport cycling rate with 140 mM extracellular sodium, but not with 40 mM (see Fig 4B in [44]). Potentially, this rate-dependent cooperativity might occurs due to changes of local submembrane ion concentrations [41] and subsequent electrostatic or electromagnetic interference between coupled and uncoupled currents at the same transporter trimeric protein. For example, increase in the inward coupled current could produce a positive shift in the intracellular voltage, which could reduce the electrical component of the transporter driving force when chloride and anionic uncoupled current are absent. However, in the presence of chloride, simultaneous, but opposite uncoupled current in the same or neighboring subunit(s) of a transporter would reverse the local voltage alteration and restore (increase) the transporter driving force and plausibly allow the second glutamate molecule to be transported.

The second option could be that the impermeable D-gluconate (which was used to replace chloride anions) blocks a second glutamate binding site. D-gluconate is an organic anion as is glutamate. Since it is present at a thousand times higher concentration than glutamate, it plausibly might compete with glutamate for a second glutamate binding site even if it might have low affinity for the site. The major glutamate binding site is considered to be associated with a conserved NMDGT motif located on transmembrane domain (TM) 7 and arginine-477 on TM8 [45]. Overall, TM7 and TM8 and the two reentrant loops HP1 and HP2 participate in the formation of the binding pocket.

It has been shown that ultra-fast glutamate application causes fast inward transient current within 130 μs [46,47]. This implies that not only negatively charged glutamate moves into the transporter (outward current), but at least two positive ions (two Na^+^ ions or Na^+^ and H^+^) also move into the transporter transmembrane electric field. Therefore, it might be possible that a second glutamate could bind to the same binding site as the first glutamate during the same transport cycle, but after 130 μs when the first glutamate moves into GLT-1 electric field and vacates the site for the second glutamate. In this case, we could assume that the affinity of the original site for the second glutamate has k_D_ = 0.17 mM, and probability of the second transport mode [3Na^+^: **2**Glu^-^: 1H^+^: (-1)K^+^] would be 50% at high glutamate concentration [27]. Therefore, the average GTS at high glutamate would be 3Na^+^: 1.5Glu^-^: 1H^+^: (-1)K^+^, with (n_Glu_ / n_e_) = 1.5 / 1.5 = 1, which is in agreement with the transport coupling slippage data.

An alternative option for the binding site for the second glutamate might be a completely different site, such as the DHK binding site, which is associated with S443 on external HP2 [48]. However, this would be a GLT-1 specific option, which is not available (with high affinity) for other glutamate transporters.

### GLT-1 cycle time is 52 ms

Previously we deliberated that the second glutamate might bind to GLT-1 only after 130 μs, when the first glutamate moves into GLT-1 electric field. However, is GLT-1 transporter cycling time sufficiently long to allow the second glutamate binding within a single transport cycle? Here we showed that GLT-1 turnover rate at 37°C and −50 mV is 19±2 s^-1^, which is unsurprisingly faster than 15 s^-1^ turnover rate at 22°C and −80 mV for human EAAT2 expressed in *Xenopus* oocytes [40]. The difference is probably due to the effect of temperature on the turnover rate (Fig 6). In addition, glutamate transporters can effectively clear synaptic glutamate within 1 ms (perhaps via glutamate binding to the transporters), and the rise time (20%–80%) of the peak of the transporter current upon ultra-fast 1 mM glutamate application was found to be in the 0.1 ms range [46,47,49]. Therefore, 52 ms GLT-1 cycle time appears to be long enough for plausible binding of the second glutamate to the transporter.

However, an extremely short (a few milliseconds) time of synaptic glutamate concentration elevation might be too short for the binding of a second glutamate to GLT-1. Yet, sometimes the synaptic glutamate is elevated for tens of milliseconds, as in the case of long bursts of spikes [26], and then synaptic GLT-1 is exposed to high glutamate concentrations that can affect the decay kinetics of excitatory transmission and possibly synaptic plasticity [50] by binding and perhaps transporting two glutamate molecules per cycle and consequently accelerating synaptic glutamate clearance. Thus, one might expect that the second GTS mode makes a supplementary contribution to synaptic glutamate clearance during long excitatory bursts.

### Physiological and pathophysiological implications of GLT-1 GTS changes

**Fig 8:**
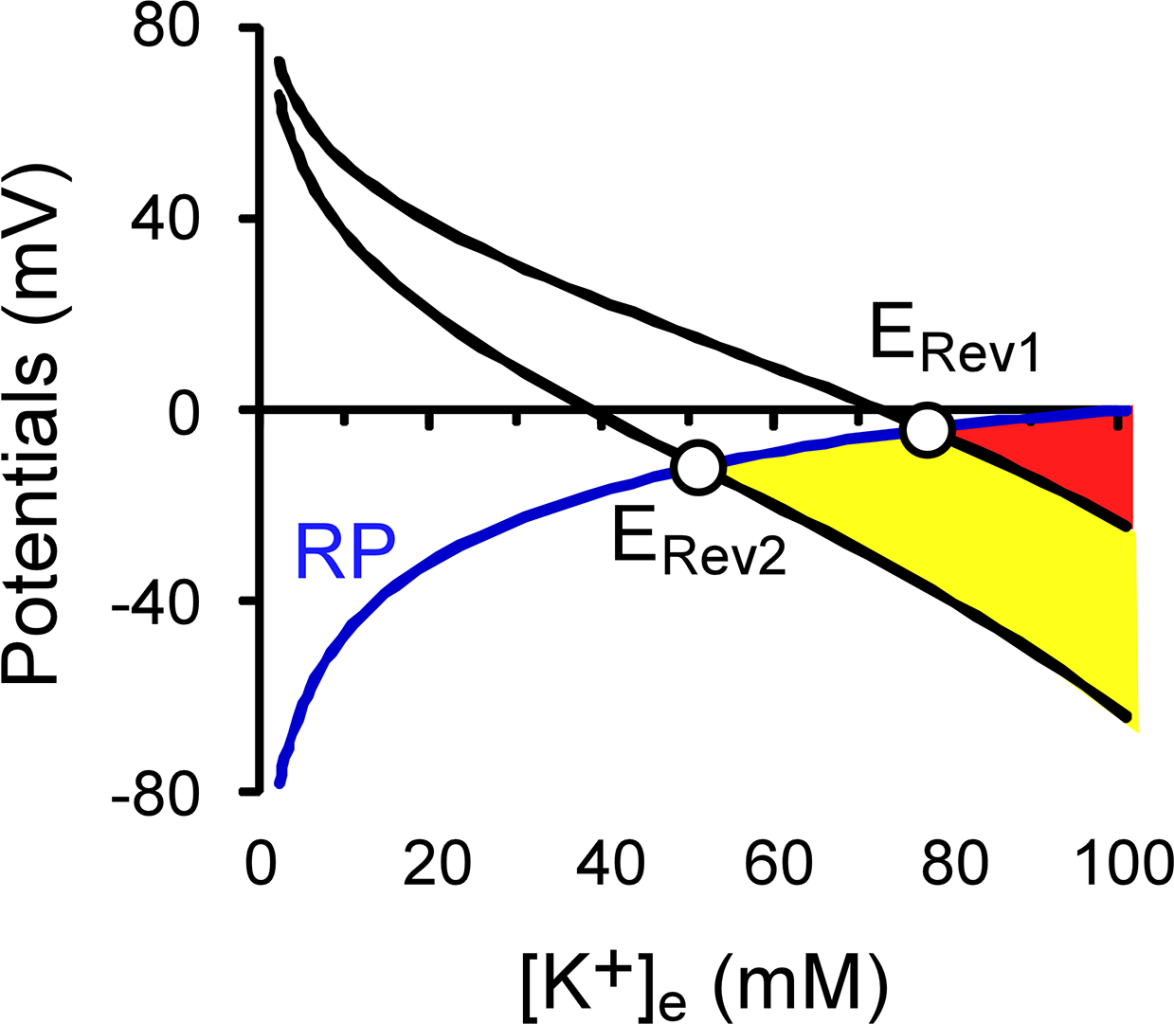
Reversal potential of GLT-1 transport during simulated ischemia depends on GTS. The resting potential (RP) depolarizes when extracellular potassium concentration rises during modeled ischemia. When GLT-1 reversal potential either with only one glutamate molecule transported per cycle (E_Rev1_), or when the 2^nd^ glutamate molecule is transported per cycle with 50% probability (E_Rev2_) are more positive than the RP, the transporter operates in normal mode and clears extracellular glutamate. The circles show the conditions of GLT-1 transport reverse at these two different transport stoichiometries.

Accurate knowledge of GLT-1 transport stoichiometry is important for a detailed understanding of the role of GLT-1 in normal synaptic transmission, as well as in the pathophysiology of stroke. During normal physiological conditions, when high glutamate concentrations are attained, the existence of the second binding site would be expected to promote glutamate clearance. However, in the excitatory synapse compromised by energy failure, glutamate transport effectively reverses its normal direction of operation and releases glutamate. This reversal occurs when the membrane potential depolarizes above the GLT-1 reversal potential, which itself depends on GTS. Specifically, GLT-1 that transports two glutamate molecules per cycle will reverse earlier than GLT-1 transporting only one glutamate (Fig 8). The influence of GTS on the GLT-1 reversal potential may be especially relevant for excitotoxicity mediated via the relatively low affinity AMPA/kainate receptors, as in global ischemia [51], and in white matter injury in the developing brain [52]. However, the genuine GTS of GLT-1 across the whole synaptic glutamate concentration range and in the presence of physiological concentrations of chloride still requires further studies.
